# Evaluating the Necessity of Deltoid Ligament Repair in Ankle Fractures: A Systematic Review and Meta-Analysis

**DOI:** 10.7759/cureus.108112

**Published:** 2026-05-01

**Authors:** Andy Suarez, Taylor Checkley, Alejandra Rinaldi, Leon Liu, Iman Fakhoury, James Ro, Christian Palacios, Mariafe Reyes, Micah Ngatuvai, Adrian Alepuz, Gary Schwartz

**Affiliations:** 1 Allopathic Medicine, Nova Southeastern University Dr. Kiran C. Patel College of Allopathic Medicine, Fort Lauderdale, USA; 2 Osteopathic Medicine, Nova Southeastern University Dr. Kiran C. Patel College of Osteopathic Medicine, Fort Lauderdale, USA; 3 Orthopedic Surgery, Texas Tech University Health Sciences Center El Paso, El Paso, USA; 4 Orthopedic Surgery, Larkin Community Hospital, Miami, USA; 5 Orthopedic Surgery, Nova Southeastern University Dr. Kiran C. Patel College of Allopathic Medicine, Fort Lauderdale, USA

**Keywords:** ankle fracture, deltoid ligament, ligament repair, medial ankle instability, meta analysis, postoperative complications

## Abstract

The deltoid ligament provides medial ankle stability and resists external rotation, excessive pronation, and abduction by anchoring the medial malleolus of the tibia to the surrounding tarsal bones. It is frequently disrupted in ankle fractures, for which optimal management remains debated. This systematic review and meta-analysis evaluated the current evidence comparing surgical repair versus non-repair of the deltoid ligament in adult patients with ankle fractures. A comprehensive search of PubMed, Embase, Web of Science, and the Cochrane Library identified prospective randomized controlled trials, with study selection conducted using Covidence by two independent reviewers, with a third reviewer resolving discrepancies. Five studies met the inclusion criteria and reported outcomes, including functional outcomes, postoperative complications, and return-to-work timelines. Meta-analysis demonstrated no significant difference in functional outcomes between repair and non-repair groups (mean difference (MD): 4.57; 95% confidence interval (CI): −3.03 to 12.17; p = 0.24) or return-to-work time (risk ratio (RR): 1.02; 95% CI: 0.65-1.60; p = 0.93); however, complication rates were significantly lower in patients undergoing deltoid ligament repair (RR: 0.43; 95% CI: 0.24-0.77; p = 0.004). While functional outcomes were comparable, deltoid ligament repair was associated with a reduced risk of postoperative complications, suggesting a potential benefit in selected patients, although larger randomized trials are needed to further define its role in ankle fracture management.

## Introduction and background

Ankle fractures are a common orthopedic injury, with an incidence of approximately 4.22 per 100,000 person-years. Many of these fractures are unstable and require surgical intervention. Additionally, a significant subset of these fractures, estimated at 10 to 40%, have a concomitant deltoid ligament tear, which further compromises medial ankle stability [1,2,3]. The deltoid ligament serves as the primary medial stabilizer of the ankle, with a recent study demonstrating that complete deltoid tears can increase eversion laxity by up to 34° and anterior translation by up to 8.6 mm [4]. When present, this associated injury may necessitate surgical repair or reconstruction in addition to bony fixation. 

Despite multiple techniques for surgically repairing torn deltoid ligaments, controversy persists regarding surgical indications, with many clinicians opting for conservative management to avoid surgical morbidity and to enable a quicker return to pre-injury activity [5]. Previous studies have compared the efficacy and outcomes of injuries treated with direct surgical repair of the ligament and those treated with closed reduction. For example, Guo et al. determined that surgical deltoid ligament repair is associated with lower complication rates compared with the non-repair group, but the pain scores between the two treatment groups were not statistically different [6]. Similarly, Hadley et al. found that surgical repair of the deltoid ligament after an ankle fracture led to fewer radiographically evident complications, but the statistical power was insufficient to draw meaningful conclusions [7].

Given these differing views, the effectiveness of deltoid ligament repair compared to nonoperative management remains unclear. This systematic review and meta-analysis aims to synthesize the current literature and evaluate functional outcomes, complication rates, and return-to-work timelines between surgical repair and non-repair in patients with ankle fractures involving the deltoid ligament.

## Review

Methods

Study Design and Search Strategy

In July 2024, a systematic review and meta-analysis were conducted across four databases (PubMed, Embase, Web of Science, and the Cochrane Library) for studies comparing the functional outcomes of patients with and without deltoid ligament repair following an ankle fracture. Search terms included "deltoid, fracture, ligament, and repair." Inclusion criteria required participants to be at least 18 years old and studies to be prospective randomized controlled trials. Case reports, retrospective studies, cadaver studies, and systematic reviews were excluded. The PRISMA (Preferred Reporting Items for Systematic Reviews and Meta-Analyses) guidelines were used to guide the conduct and reporting of the review [8,9].

Study Selection

Following the search of the databases, studies were uploaded to the Covidence (Covidence systematic review software, Veritas Health Innovation, Melbourne, Australia) data extraction tool, where duplicate articles were automatically removed. This process was reviewed to ensure that no errors occurred. Two independent reviewers screened the deduplicated articles based on their titles and abstracts. Studies that were deemed irrelevant or that followed an alternate study design were excluded. The remaining studies underwent full-text screening by two independent reviewers using the predetermined inclusion and exclusion criteria. Each reviewer worked independently, and article selection was blinded. Discrepancies in article selection were resolved through discussion with a third reviewer.

Data Extraction and Risk of Bias

Data extraction was performed by two independent reviewers utilizing templates provided by Covidence. The templates were used to collect data regarding the articles’ background, methods, population characteristics, interventions, and outcomes. Some studies additionally reported outcomes specific to their unique research objectives; however, those outcomes were not included in this meta-analysis. Extraction was performed on metrics such as the American Orthopedic Foot and Ankle Hindfoot Score (AOFAS) [10], which was used to assess both patient-reported and clinician-assessed functional outcomes. AOFAS requires patients to indicate their level of function, ranging from a minimum score of 0 (completely impaired function) to a maximum of 100. Additional outcomes included postoperative complications and time to return to work.

Risk of bias was assessed using a meta-analysis tool, Review Manager (RevMan) version 5.4.1 (The Cochrane Collaboration, 2020). Various parameters of each study were scored by two independent reviewers using a scale of low to high likelihood of bias. The parameters assessed included aspects of study design, such as blinding, selective reporting, and random sequence generation.

Data Analysis

Extracted data were pooled and analyzed using Review Manager software (RevMan 5.4.1). Continuous outcomes, such as return to work, were pooled only when reported in comparable units (weeks). The mean difference (MD) was calculated, and standard deviations (SD) were used to calculate variance. For dichotomous outcomes, risk ratios (RR) were estimated, and the Mantel-Haenszel statistical method was applied. Point estimates for all measures were calculated with their corresponding 95% confidence intervals (CI). All tests were conducted with a significance threshold of 0.05. Heterogeneity was assessed by calculating the I² index, with values greater than 50% representing moderate to high heterogeneity. A random-effects model was used when heterogeneity was greater than 50%, while a fixed-effects model was used when heterogeneity was less than 50%.

Results

Study Selection

**Figure 1:**
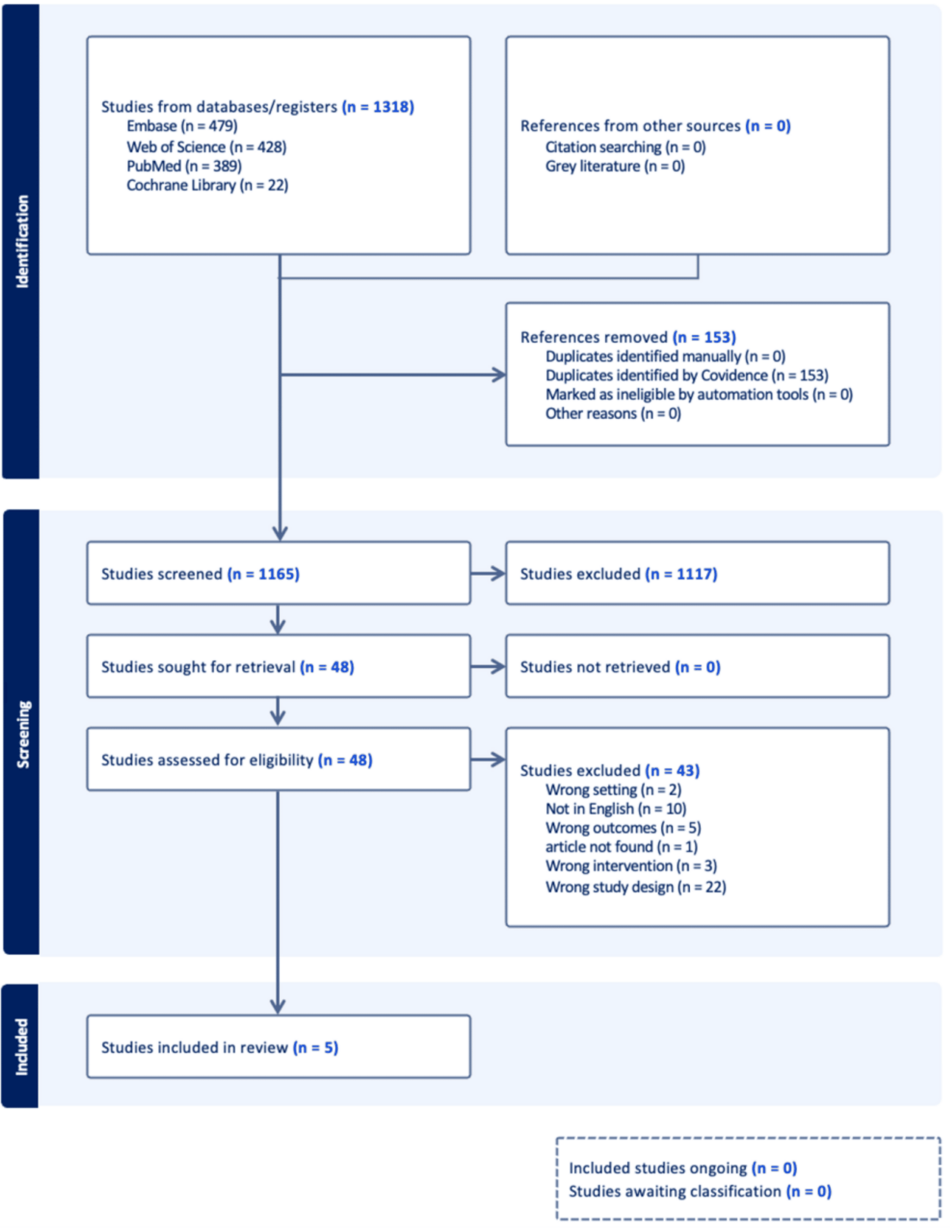
PRISMA flow diagram depicting the study selection [9] PRISMA: Preferred Reporting Items for Systematic Reviews and Meta-Analyses

A total of 1,318 results were generated using the terms “deltoid,” “fracture,” “ligament,” and “repair.” After studies were deduplicated, 1,165 studies underwent title and abstract screening. After the resulting studies underwent full-text screening, a total of five studies were deemed eligible to be included in the final data analysis of this meta-analysis. Figure 1 presents the PRISMA flow diagram depicting the selection of studies. Table 1 provides a summary of the characteristics of the included studies.

**Table 1:** Summary of the included studies [11,12,13,14,15] RTC: randomized controlled trial

Study	Type	Participants	Interventions	Outcomes	Results
Strömsöe et al., 1995 (Norway) [11]	RCT	N = 50; no repair: 25, deltoid repair: 25	Repair of the deltoid ligament vs. no repair	Postoperative complications; return to work	No significant difference in postoperative complications or return to work
Gu et al., 2017 (China) [12]	RCT	N = 40; no repair: 20, deltoid repair: 20	Repair of the deltoid ligament vs. no repair	Functional outcomes; postoperative complications	Significantly fewer postoperative complications with repair; no significant difference in overall postoperative complications
Rungprai et al., 2017 (Thailand) [13]	RCT	N = 41; no repair: 20, deltoid repair: 21	Repair of the deltoid ligament vs. no repair	Return to work	No significant difference in return to work
Sun et al., 2018 (China) [14]	RCT	N = 41; no repair: 13, deltoid repair: 28	Repair of the deltoid ligament vs. no repair	Functional outcomes; postoperative complications	No significant difference in functional outcomes or postoperative complications
Choi et al., 2022 (Korea) [15]	RCT	N = 34; no repair: 15, deltoid repair: 19	Repair of the deltoid ligament vs. no repair	Functional outcomes	No significant difference in functional outcomes

Outcomes

**Figure 2:**
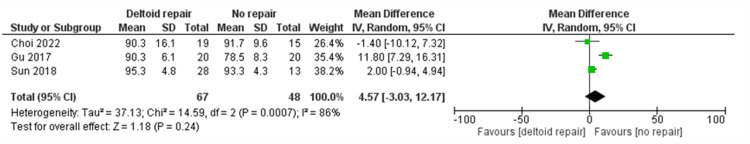
Forest plot comparing functional outcomes [12,14,15] SD: standard deviation; CI: confidence interval

Three studies reported on the functional outcome of patients using the AOFAS. Data from a total of 115 participants revealed an MD of 4.57; however, this was not significant (95% CI: -3.03 to 12.17, p = 0.24; Figure 2).

**Figure 3:**
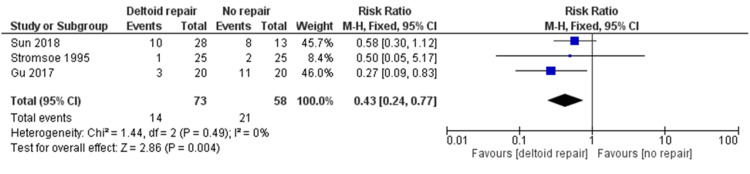
Forest plot comparing postoperative complications [11,12,14] CI: confidence interval

Three studies reported on the rate of postoperative complications experienced by patients with and without deltoid ligament repair. Complications included surgical site infection, bleeding, and pulmonary infection. Patients in the non-repair group had a significantly increased risk of experiencing a complication when compared to the deltoid ligament repair group (RR 0.43; 95% CI: 0.24 to 0.77; p = 0.004; Figure 3).

**Figure 4:**
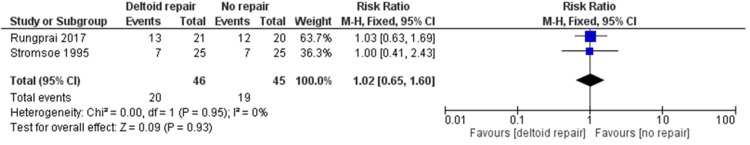
Forest plot comparing time to return to work [11,13] CI: confidence interval

Time taken to return to work was reported on by two included studies. Pooled data from a total of 91 participants revealed an RR of 1.02. These results, however, did not reach statistical significance (RR: 1.02, 95% CI: 0.65 - 1.60, p = 0.93; Figure 4).

Risk of Bias

**Figure 5:**
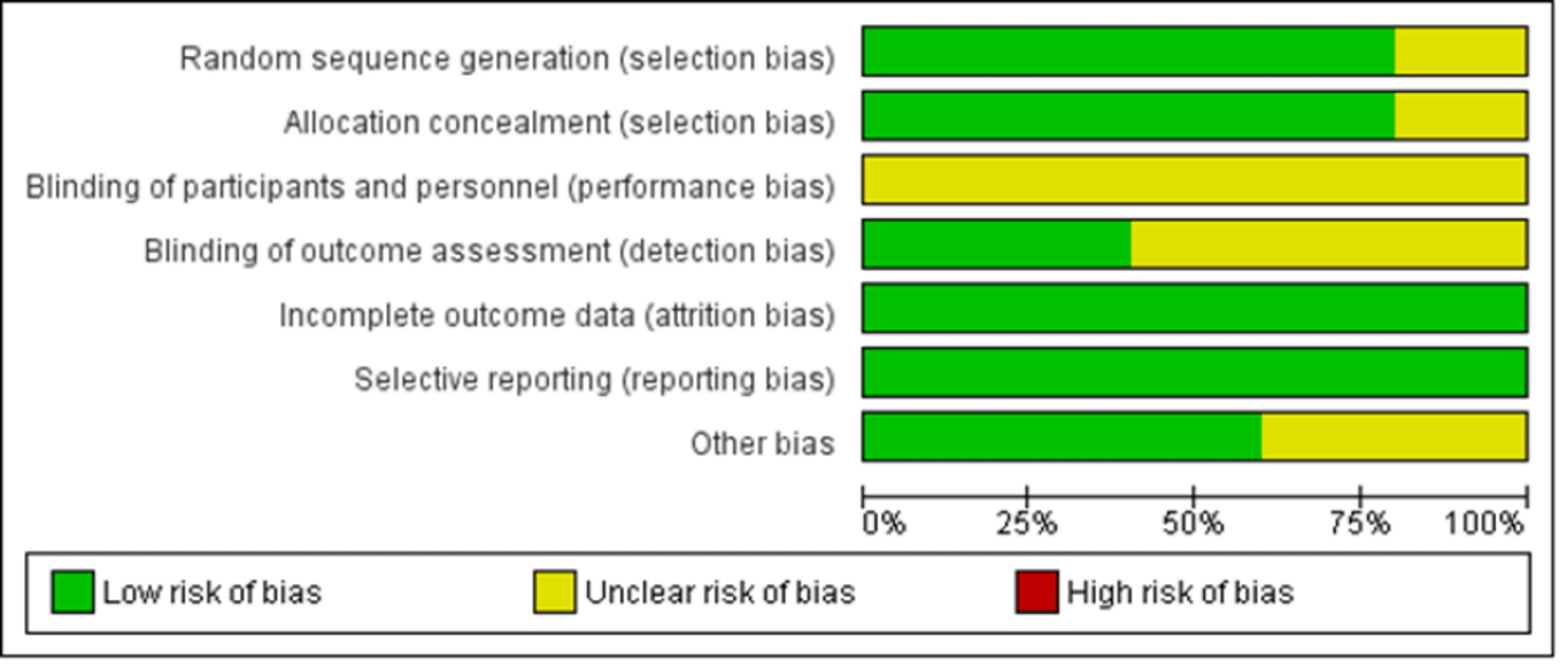
Risk of bias graph [11,12,13,14,15]

**Figure 6:**
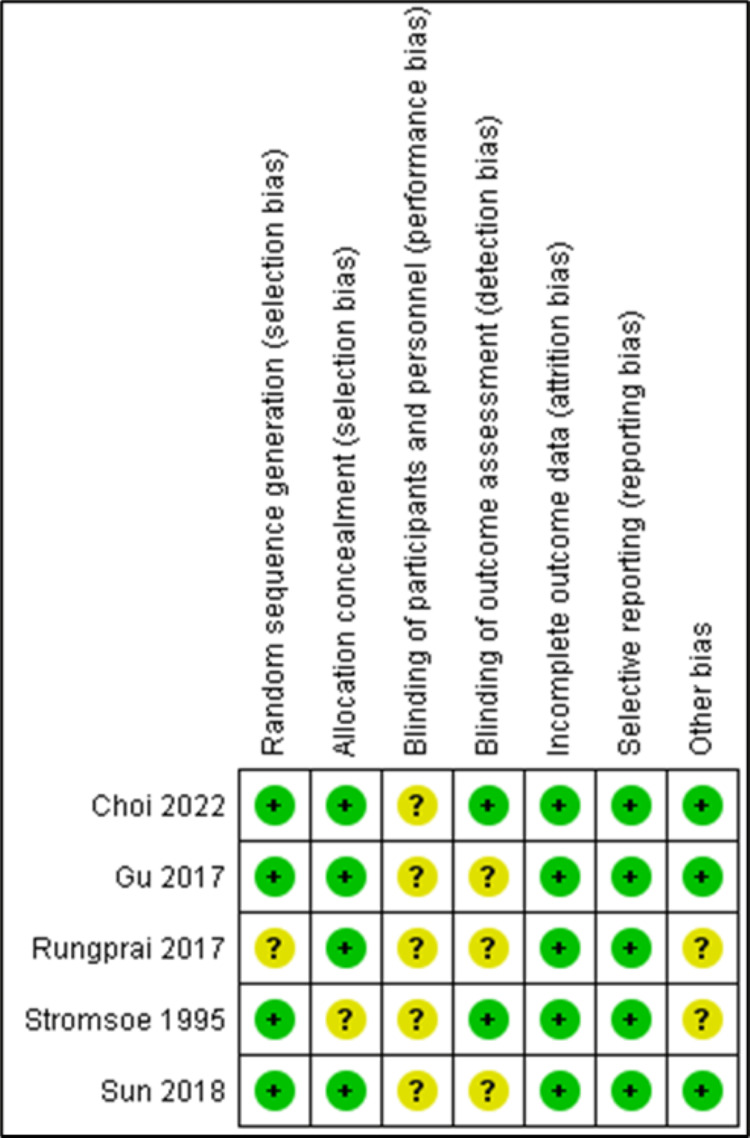
Risk of bias summary [11,12,13,14,15]

Risk of bias was assessed for all five included studies. The primary source of bias among our included studies was related to the blinding of participants and personnel, consistent with the methodology and hypothesis of the studies revolving around a surgical intervention. A few articles also had unclear bias in the blinding of outcome assessment and other types of bias (Figures 5, 6).

Discussion 

The meta-analysis indicated no statistically significant difference in AOFAS scores or return-to-work time between repair and non-repair groups. The analysis also revealed that pooled complication rates were significantly lower in the surgical repair group. This likely affirms that surgical intervention bolsters the stability of the damaged deltoid ligament throughout the healing process, reducing the need for additional corrective surgery [16]. 

The findings from this analysis are generally consistent with existing literature. A similar meta-analysis by Guo et al. [6] also indicated no statistical difference in AOFAS scores across the two treatment methods, a finding that is further aligned with standalone randomized controlled trials [17]. This report, however, also reported complication rates to be higher in the non-repair group [6], a finding that is consistent with the results of our present report as well as those of other meta-analyses [7]. Similarly, a study by Woo et al. reported that although the clinical outcomes were not significantly different between the two groups, they obtained a more favorable final follow-up medial clear space (MCS) in the deltoid repair group. Particularly when accompanied by a syndesmotic injury, the final follow-up MCS and the clinical outcomes were better in the deltoid repair group [18]. An additional study also showed radiographic outcomes with a statistically significant decrease in the medial clear space and decreased malreduction rates postoperatively in the deltoid ligament repair groups [19]. The consistency of our findings with those of the existing literature supports the claim that surgical repair of the deltoid ligament offers benefits in terms of reduced complication rates but not in AOFAS scores. 

Our study demonstrated a lower reoperation rate in patients who underwent deltoid ligament repair compared to those who did not, similar to other studies, which showed reduced reoperation rates due to instability in patients undergoing deltoid ligament repair [20]. Therefore, the deltoid ligament may provide a benefit in reducing postoperative complications. This finding, however, should be interpreted with caution, as several confounding factors may account for this difference. It is possible that patients who did not undergo repair presented with more severe fracture patterns or non-repairable deltoid ligament injury, inherently predisposing them to higher complication rates. Alternatively, the decision to repair the deltoid ligament may serve as a proxy for surgeon experience, with the possibility that the most experienced surgeons were also more likely to perform deltoid ligament repair. Given the limitations of the current literature and available evidence, we recommend that surgeons routinely evaluate for deltoid ligament insufficiency in the setting of ankle fractures and strongly consider repair in cases of confirmed incompetence.

Our study has several limitations, including a relatively small sample size; 115 patients were measured for the AOFAS score, and 91 were assessed for return to work. Both of these metrics demonstrated no statistical significance, which may be attributed to the underpowered nature of the analysis. Next, there was significant heterogeneity of metrics used to assess outcomes reported in these cases. For example, postoperative ankle stability, an important characteristic of Danis Weber classifications, was not measured in several previous studies [5]. Methodologies for evaluating ankle stability also remained subjective across the literature. For instance, De Souza et al. implemented a 100-point score to describe various metrics such as pain, function, range of motion, and radiographic findings [21]. Although a score of 80 out of 100 was considered a satisfactory result in this study, this threshold lacks standardization across other reports [7]. The variability in fracture type, surgical techniques, rehabilitation protocols, and functional scoring further limits the generalizability of findings.

Additionally, unclear blinding of participants and outcomes introduces the possibility of performance and detection bias. For example, surgeons may have patients complete different postoperative protocols, such as time to weight bearing or method of immobilization, depending on whether the deltoid ligament was repaired, and therefore, outcomes may instead be related to differences in postoperative protocols rather than whether the deltoid was fixed. This reduces confidence in the suggested correlation between deltoid repair and reduced complication rates and may have resulted in an overestimation of its potential benefit. Regardless, the study is an important contribution to the literature by providing a quantitative assessment of parameters comparing surgical treatment of the deltoid ligament versus non-repair treatment. Further large-scale, randomized controlled trials are needed with standardized scoring systems and longer follow-up durations.

## Conclusions

Our findings showed that while functional outcomes may not differ considerably between the treatment modalities, surgical fixation of the deltoid ligament demonstrates a protective association against common postoperative complications. This meta-analysis of five prospective randomized controlled trials evaluating outcomes following deltoid ligament repair in ankle fractures found no statistically significant differences in functional recovery or return to work timelines between patients undergoing ligament repair and those managed without repair. However, adverse postoperative complications, including incision site infection, bleeding, and pulmonary infection, revealed a statistically significant reduction in patients who underwent deltoid ligament repair. Future randomized studies with larger sample sizes and standardized outcomes are needed to refine patient selection criteria and establish evidence-based guidelines for the operative management of deltoid ligament injuries in ankle fractures.
